# How disentangled sense of agency and sense of ownership can interact with different emotional events on stress feelings

**DOI:** 10.1186/s41155-017-0071-y

**Published:** 2017-08-23

**Authors:** Wei Chen, Jing Zhang, Yanyan Qian, Qiyang Gao

**Affiliations:** 1 Department of Psychology, Shaoxing University, Shaoxing, China; 2 Institute of Psychological Health, Hangzhou Dianzi University, Hangzhou, China; 3 Leiden Institute for Brain and Cognition, Leiden University, Leiden, The Netherlands; 4 School of Psychology, Nanjing Normal University, Nanjing, China

**Keywords:** Sense of agency, Sense of ownership, Virtual hand illusion, Emotional events, Anxiety, Attributional style, Empathy ability

## Abstract

We used the virtual hand illusion paradigm to study how sense of agency and sense of (body) ownership can interact with different emotional events on stress feelings. Converging evidence for at least the partial independence of agency and ownership was found. For instance, sense of agency was a better predictor of individual anxiety levels than sense of ownership and males showed stronger effects related to agency—presumably due to gender-specific attribution styles and empathy skills. Moreover, agency and ownership also interacted with emotional events and led to different anxiety levels. Taken together, our findings suggest that the disentangled sense of agency and sense of ownership can interact with different emotional events and influenced stress feelings more in threatening situations than awarding ones.

## Background

*“Were the size of my hand, such as its length, width, span, etc. given to me as inalterably fixed, the attempt at empathy with any hand having different properties would have to fail because of the contrast between them. But actually empathy is also quite successful with men's and children's hands which are very different from mine, for my physical body and its members are not given as a fixed type, but as an accidental realization of a type that is variable within definite limits. On the other hand, I must retain this type. I can only empathize with physical bodies of this type; only them can I interpret as living bodies. This is not yet an unequivocal limitation.”*

—Edith Stein, 1916

The phenomenon of rubber hand illusion was first reported by Botvinick and Cohen (Botvinick & Cohen, 1998) in which a rubber hand was placed in front of the participants while their own hands were hidden from sight. As long as there were synchronous touches that existed on both rubber hand and real hand, a perceptual illusion would be felt. Besides recognizing the fake model hand as being part of their own body, participants also reported that they felt as if the touch they sensed originated from the location on the rubber hand where they saw the brush touching the rubber hand, rather than from their real hand (Makin, Holmes, & Ehrsson, 2008). Furthermore, recent studies show below rubber hand illusion implicate that the brain is capable of integrating “natural” visual input and direct cortical-somatosensory stimulation to create the multisensory perception that an artificial limb belongs to one’s own body (Tsakiris, 2017; Collins et al. 2017).

This method is widely used, with minor alternations, to induce illusions and investigate individual self-perception of the body which is critically important for conscious experience of the self (Folegatti, Farnè, Salemme, & Vignemont, 2012; Germine, Benson, Cohen, & Hooker, 2013; Jenkinson, Haggard, Ferreira, & Fotopoulou, 2013; Ocklenburg, Peterburs, Rüther, & Güntürkün, 2012). Sense of agency and sense of ownership are considered to be two main aspects of minimal self which according to Gallagher is a basic, immediate, or primitive “something” that we are willing to call a self (Gallagher 2000a, b) and thus enables us to capture the most primitive sense of self (Gallese & Sinigaglia, 2010). There are numerous empirical and theoretical researches using the rubber hand illusion paradigm to study the distinction between sense of agency and sense of ownership in order to explain how we perceive ourselves.

Sense of agency refers to the pre-reflective experience or sense that I am the cause or author of the movement, while sense of ownership is the pre-reflective experience or sense that a limb is part of one’s body (Tsakiris, Schuetz-Bosbach, & Gallagher, 2007; Tsakiris, 2017). In normal experience of voluntary or willed action, sense of agency and sense of ownership coincide and are indistinguishable; in the case of involuntary movement, however, it is quite possible to distinguish these two senses (Gallagher, 2000a, b). The distinction between sense of agency and sense of ownership has attracted considerable interests in various fields including psychology, philosophy, and cognitive science (Blakemore, Wolpert, & Frith, 2002; Marcel, 2003; Tsakiris & Haggard, 2005; Chen, 2016). Although different methodologies we may find in different disciplines, there is a growing consensus on this division between sense of agency and sense of ownership. According to experimental research on normal subjects, sense of agency for action is based on that which precedes action and translates intention into action while sense of ownership for motor action can be explained in terms of ecological self-awareness built into movement and perception (Gallagher, 2000a, b). Zhang and Chen (2016) adopted distance reference as a new factor to investigate the plasticity of the body image. Their study found that distance reference frame influenced peoples’ perception of their own body. The size of the ownership illusion varied as a function of relative rather than absolute location of the virtual hand. The result suggests a considerable degree of plasticity of the body image underlying our body ownership. Haggard, by considering the logic of involuntary movement where there is a sense of ownership but no sense of agency, suggested that in ordinary voluntary movement, the sense of ownership is generated by sensory feedback, while the sense of agency is generated by or at least linked to the motor commands sent to the muscles and the accompanying efferent copy that is internally processed within the predictive models of the motor system (Haggard, 2005; Tsakiris, Schuetz-Bosbach & Gallagher, 2007).

There have been several studies which focused on the differences between sense of agency and sense of ownership that clearly dissociated these two experiences. For example, Santo and Yasuda, by manipulating the discrepancy between the intended and actual consequences of actions, found that a discrepancy between predicted and actual feedback had significant impacts on sense of agency but no effects on sense of ownership (Sato & Yasuda, 2005). Tsakiris and colleagues, using a video-screen-based setup, discovered that the types of proprioceptive drift differed among different situations. They found localized proprioceptive drifts for tactile and passive stimulation but not for active movement, which means a purely proprioceptive sense of ownership is local and fragmented but the motor sense of agency integrated distinct body parts into a coherent, unified awareness of the body (Tsakiris, Prabhu, & Haggard, 2006). Longo and colleagues, using a 27-item questionnaire which was designed based on qualitative research with five participants, estimated participants experience after they were stroked synchronously or asynchronously. Their results suggested that ownership and agency are two of those subcomponents of embodiment (Longo et al., 2008). Kalckert and Ehrsson, using a moving rubber hand setup, varied the relative timing of the figure movements, the mode of movement, and the position of the model hand. The results that asynchrony eliminated both agency and ownership and passive movements abolished the sense of agency but not the ownership while incongruent positioning the model hand diminished ownership but not agency provided evidence for a double dissociation of sense of agency and sense of ownership, suggesting they may represent distinct cognitive processes (Kalckert & Ehrsson, 2012). There are also studies providing empirical evidence about such dissociation in terms of neural correlates. Tsakiris et al., using fMRI, investigated the sensory and motor aspects of body-representation in the brain. Their findings supported the idea that agency and body ownership are qualitatively different experiences, triggered by different inputs, and recruiting distinct brain networks (Tsakiris, Longo, & Haggard, 2010).

Besides rubber hand illusion and its revised version, virtual hand illusion is another way to induce body perception illusion. In the experiment of virtual hand illusion, participants sit in front of a screen where a virtual 3D image of the virtual hand would be presented while having tactile stimulation on their real hidden hand. It is indicated that the way of inducing virtual hand illusion can achieve the same effect as what rubber hand illusion did. In other words, imposing the same tactile stimulation on both the virtual hand on the screen and the real hand which is hidden from view can let the participants feel the similar experience to that under rubber hand illusion condition (Ma & Hommel, 2013; Zhang & Chen, 2016). Experiment showed that by simply manipulating the temporal delay between participants’ real movement and the movement of the virtual hand on the screen, a virtual hand illusion can be induced even in the absence of tactile stimulation (Sanchez-Vives et al., 2010). Slater et al. found that there were reliable correlations between the impression of hand ownership and hand-related electromyography (EMG) activation, suggesting a connection between perceived ownership and action control (Slater, Perez-Marcos, Ehrsson, & Sanchez-Vives, 2008).

No matter what method is used, researches in this area paid more attention to the relationship between sense of agency and sense of ownership rather than how sense of agency and sense of ownership can affect our higher cognition, such as emotional experiences (Guterstam, Abdulkarim, & Ehrsson, 2015; Christensen, Yoshie, Di, & Haggard, 2016). Though there have been already some studies using this paradigm of virtual hand illusion to investigate the relationship between sense of ownership and the affective resonance in facing with different kinds of emotional events. Yuan and Steed designed an experiment to measure skin conductance responses (SCR) to what they considered threats to a virtual hand and found similar elevations as with rubber hands. Participants were asked to play games in virtual environment by operating the hand of an avatar. During the game, a virtual lamp would fall on the virtual hand operated by the participants at some point, which induced a reliable increase in SCR. They placed the hand with an arrow as the control condition which produced significantly less increase in SCR. Taken together, they suggested that people emotionally “care” about what they perceive as being a part of their body but not, or not so much, about what they perceive as belonging to the body of someone else (Yuan & Steed, 2010). However, Ma and Hommel thought that two aspects of Yuan and Steed’s study might help explaining this seeming discrepancy. For one, they did not use the standard synchronization technique to induce different degrees of body ownership. For another, the threatening event merely consisted of a virtual lamp falling on the virtual hand. Even though the contact between the lamp and the hand was clearly visible to the participant, it is difficult to judge from the visual display how much pain. Ma and Hommel adopted the standard synchronization technique to induce the illusion of ownership and replaced the falling of a virtual with a knife. Their findings suggested that ownership was stronger if the virtual hand moved synchronously with the participant’s own hand, but this effect was independent from whether the hand was impacted or threatened. In other words, in the face of threats, affective resonance was independent of synchronicity (Ma & Hommel, 2013).

However, we think there are still some problems that need to be dealt with. As two main aspects of minimal self, sense of agency and sense of ownership are connected with each other in a complex way. Two common models are existing in terms of the relations of senses of agency and ownership. In an “additive” model, agency and ownership are strongly related, while an“independent” model holds agency and ownership differ in experiences quanlitatively (Tsakiris, Longo, & Haggard, 2010). Even though they have been approved to be driven by different kinds of information and related to different psychological functions (Tsakiris, 2017), it is necessary to consider both of these two experiences when studying how bodily states can affect emotional feelings. The available evidence can be taken to suggest that ownership and agency are strongly related to affective reactivity (Ma, Lippelt, & Hommel, 2017); however, it is more common to use emotional variables as stimuli to investigate their impact on agency and ownership but not vice versa. In the present study, we tried to investigate how different situations of sense of agency and sense of ownership could interact different emotional events in terms of stress feelings.

We carried out two experiments. Experiment 1 aimed to find out how sense of agency and sense of ownership can affect participants’ anxiety after they performed certain type of task in terms of different genders. Experiment 2 further divided the tasks into two types (positive and negative feedbacks, respectively) to study how disentangled sense of agency and sense of ownership can interact with different emotional events on stress feelings.

## Method

### Experiment 1

#### Participants

The participants were 96 undergraduate students (48 females, 48 males) from four universities in Zhejiang and Nanjing, China, who were unfamiliar with rubber/virtual hand illusion and took part in this study voluntarily. The age of the participants ranged between 18.24 and 29.67 (*M* = 21.32, SD = 2.54). All the participants were right handed with normal naked or corrected visual acuity. Ethical approval for this study was obtained from the relevant university ethics committee, and informed written consent was obtained from all subjects.

#### Material

**Fig. 1:**
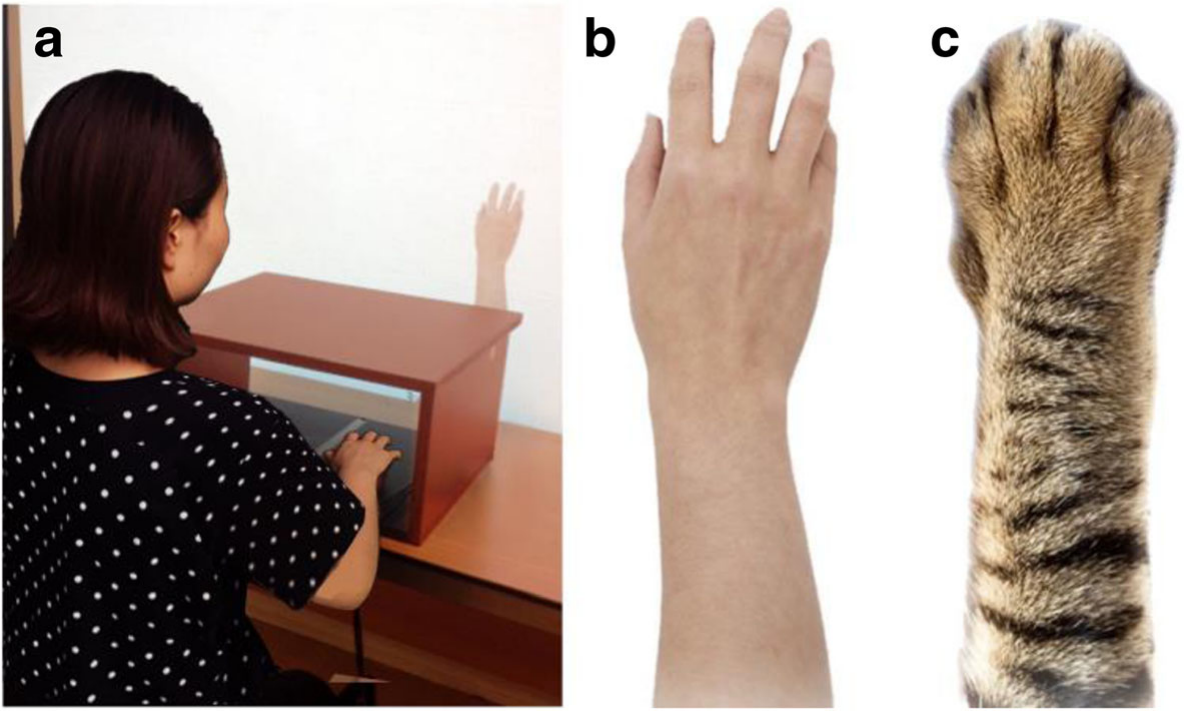
Experiment and virtual images. **a** experimental facilities. **b** human hand. **c** cat's claw

The study was performed in a virtual environment, which was programmed by VB.NET. A virtual human hand or cat claw was presented on the screen (see Fig. 1b, c). The mouse was placed in front of the screen but shielded by a special box.

**Fig. 2:**
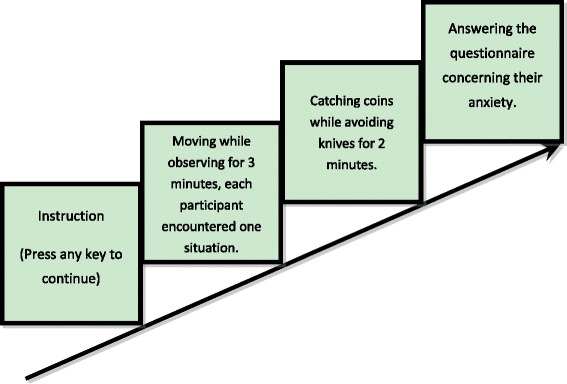
Setup of experiment 1

This experiment was composed of two parts. In the first part, a virtual human hand or cat claw was presented on the screen moving either strictly in accordance with the movement of the mouse or slightly delayed (350 to 500 ms), and the participants were asked to observe the movement of the virtual human hand/cat claw while moving the mouse with their right hands for 3 min (see Fig. 1a). According to our previous study, the treatment as in part 1 can be used to induce sense of agency and sense of ownership, respectively. The synchronicity manipulation can induce sense of agency, while the modality had a strong effect on sense of ownership. The second part appeared right after the end of the first part. In the second part, there were knives and coins falling down the screen, and the participants needed to catch coins as well as avoid knives. After finishing the task, the participants were asked to answer some questions which we adopted from the State-Anxiety Inventory (S-AI) to evaluate their anxious level (see Fig. 2).

#### Questionnaire

We adopted the reverse scoring questions from the S-AI to access participants’ anxiety level after the treatment of experiment 1 (see Table 1). The S-AI is a self-report questionnaire that consists of 20 items for measuring state anxiety (Spielberger et al. 1983). The Chinese version of the S-AI demonstrates satisfactory reliability and validity with regard to Chinese populations (Shek, 1988; Zheng et al. 1993). For the State-Anxiety Inventory (STAI), Cronbach’s alpha coefficient for internal consistency in our sample is acceptable (*α* = 0.87).

**Table 1:** Questions from State-Anxiety Inventory

1	I feel calm.	11	I feel self-confident.
2	I feel safe, secure.	12	I feel nervous, irritable.
3	I feel tense, nervous.	13	I feel scared, alarmed, afraid.
4	I feel stressed.	14	I feel uncertain.
5	I feel peaceful, good about myself.	15	I am relaxed, at ease.
6	I feel upset, overwhelmed.	16	I am satisfied.
7	I worry over possible misfortunes.	17	I am anxious, worried.
8	I feel happy.	18	I feel disconcerted, disoriented.
9	I feel frightened.	19	I feel collected, composed.
10	I feel at ease.	20	I feel pleasant, in a good mood.

#### Procedure

There were three factors in this experiment: synchronicity (synchronous vs. asynchronous), modality (human hand vs. cat claw), and sex (female vs. male). The purpose of this experiment was to study whether different situations of agency and ownership will affect participants’ anxiety or not after they performed the rewarding/punishing task, namely how sense of agency and ownership will influence people’s feeling when facing task which involved emotional feedbacks. An early consistent finding of Witkin’s investigation of individual differences in perception was that women tend to be more field dependent than men (Witkin et al., 1962). And previous study also confirmed that women tend to use an external strategy of explanation while men orient themselves towards something internal (Sørensen, 2005). Therefore, we added sex as another factor, which made the experiment a three-factor between-subjects design.

The movement between the virtual image and the participant’s real hand was either synchronous or asynchronous, and the virtual image was either a human hand or a cat claw, except for the participants who needed to perform a catching/avoiding task after moving their real hands and watching the movements of the virtual image on screen for 3 min. The participants saw virtual coins and knives coming down from the top of the screen, and what they needed to do was to catch as many coins as they can and meanwhile avoid the cut of the falling knives. The scores of their performance appeared on the top right corner of the screen during the whole task. Catching a coin or avoiding a knife would add a point while losing a coin or being cut by a knife would lose a point. There were eight situations in this experiment. Each participant encountered one. They were asked to play this catching/avoiding game for 2 min. At the end of the task, there would be a message printed on the screen which told them the results of their performances. After the experiment, the participants needed to fill out the S-AI.

### Results and discussion

Considering that anxiety level may not be so sensitive to measure, especially for those statements describing their anxious states, so we only calculated the results of those reverse scoring statements (No. 1, 2, 5, 8, 10, 11, 15, 16, 19, 20) of the S-AI. Thus, the anxiety score of this study was the sum of standard scores for question 1, 2, 5, 8, 10, 11, 15, 16, 19, and 20.

**Fig. 3:**
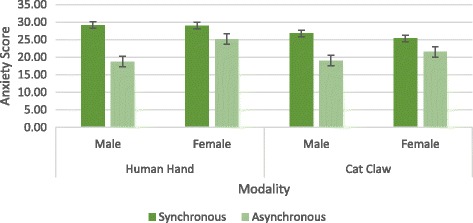
The results of experiment 1

The mean ratings for anxiety were submitted to a 2 × 2 × 2 ANOVA with the three factors which are synchronicity (synchronous vs. asynchronous), modality (human hand vs. cat claw), and sex (female vs. male) (see Fig. 3). There were significant main effects of the type of synchronicity and modality (*F* (1, 95) = 48.62, *p* < 0.001, *η* ^2^_*p*_ = 0.55 and *F* (1, 95) = 6.35, *p* < 0.014, *η* ^2^_*p*_ = 0.20, respectively). The participants showed a stronger sense of anxiety for synchronous (*M* = 27.52, SD = 4.43) than asynchronous views (*M* = 21.13, SD = 5.31) and also a stronger sense of anxiety for human hand (*M* = 25.52, SD = 5.66) than cat claw views (*M* = 23.19, SD = 5.86). From the result of pilot study, we know synchronicity affected sense of agency and modality affected sense of ownership, which means the more sense of agency and sense of ownership during the task, the stronger the sense of anxiety. The effect of synchronicity on anxiety was more dramatic than that of modality which indicated the feeling of controlling something would put more pressure on participants than the feeling of owning something.

The interaction between synchronicity and sex was also significant (*F* (1, 95) = 8.03, *p* = 0.006, *η* ^2^_*p*_ = 0.64). This interaction effect suggested there were stronger effects related to sense of agency for male (*M* = 28.00 for synchronous and *M* = 18.92 for asynchronous) when compared with female participants (*M* = 27.17 for synchronous and *M* = 23.33 for asynchronous). There were similar results when we looked into the effects of modality on anxiety in terms of sex. There was also a difference between males (*M* = 23.96 for human hand and *M* = 22.96 for cat claw) and females (*M* = 27.08 for human hand and *M* = 23.42 for cat claw). Stronger effects related to sense of ownership was found, although the difference is not significant (*F* (1, 95) = 2.072, *p* = 0.154). These effects differed between the sexes. Sense of agency influenced male more than female while sense of ownership had the vice versa effect. Males seemed to prefer inner attribution which made them feel more anxiety about their performance during the catching/avoiding task whenever they had a strong sense of agency. This result is consistent with a previous study showing that compared to the group of men, women use an external strategy of explanation (Sørensen, 2005). A number of studies had substantially confirmed such individual differences in perception that women tend to be more field dependent than men (Witkin et al., 1962). Because women are more likely to do external attribution while men prefer internal attribution (Rim, 1990; Wang et al. 2013), when dealing with the emotional feelings aroused by the rewarding/punishing task, the attribution style of female helped them to ease.

Experiment 1 indicated that sense of agency and sense of ownership had significant effects on people’s anxiety after performing the task which involved positive and negative feedbacks. Interestingly, the results of experiment 1 also showed the effects of agency and ownership on perceived anxiety were different, which suggested that the informational bases for these two judgments do not entirely overlap, showing a discrepancy between ownership and agency (Kalckert & Ehrsson, 2012; Kalckert & Ehrsson, 2014). Also, previous studies showed that people experience more anxiety in the face of threat targeting a virtual effector that they also perceive more ownership for (Ma & Hommel, 2015; Zhang & Hommel, 2016); in order to know more about how positive and negative events can interact with sense of agency and sense of ownership in terms of anxiety, we performed experiment 2 which was very similar with experiment 1 except each participant was assigned to only one kind of task, catching coins or avoiding knives.

### Experiment 2

#### Participants

The participants were 96 undergraduate students (48 females, 48 males) from four universities in Zhejiang and Nanjing, China, who were unfamiliar with rubber/virtual hand illusion and took part in this study voluntarily. The age of the participants ranged between 17.89 and 27.90 (*M* = 21.04, SD = 2.34). All the participants were right handed with normal naked or corrected visual acuity. Ethical approval for this study was obtained from the relevant university ethics committee, and informed written consent was obtained from all subjects.

#### Material

**Fig. 4:**
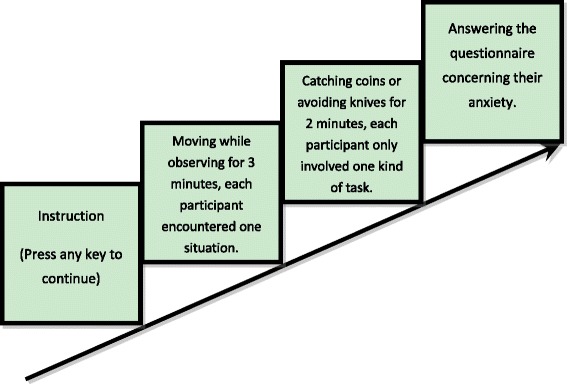
Setup of experiment 2

Experimental setup was almost the same as of experiment 1, except for the content of the task. In experiment 1, after 3-min virtual hand illusion treatment, the participants were asked to perform a 2-min task which was catching coins while avoiding knives. However, in this experiment, there was only one type of task for each participant, which means for each participant, what he or she encountered was either the catching coins task or the avoiding knives task. After the end of the task, the participants were asked to fill out the S-AI (see Fig. 4).

#### Questionnaire

The same questionnaire as in experiment 1 was used in this experiment.

#### Procedure

There were three factors in this experiment: synchronicity (synchronous vs. asynchronous), modality (human hand vs. cat claw), and event (catching coins vs. avoiding knives). Because the difference between the sexes was not significant in experiment 1, for this experiment, we did not consider sex as a factor any more. The purpose of this experiment was to study how different tasks will interact with different situations of agency and ownership, namely how sense of agency and ownership will influence people’s feeling when facing different emotional events.

The procedure was very similar to that in the experiment 1, the movement between the virtual image and participant’s real hand was either synchronous or asynchronous, the virtual image was either a human hand or a cat claw, and the task participants needed to perform was either catching coins or avoiding knives. After the beginning of the experiment, the participants were asked to move their hand and watch the movement of the virtual image for 3 min, which was meant to induce their sense of agency or sense of ownership. After that, they would either see coins or knives falling on the screen, and what they needed to do was to catch as many coins or avoid the cut of the falling knives. The same as in experiment 1, there were scores of their performance displayed on the top right corner of the screen during the task. Catching a coin or avoiding a knife would add a point while losing a coin or being cut by a knife would lose a point. There were eight situations in this experiment. Each participant encountered one. At the end of the task, there would be a message printed on the screen which told them the results of their performances. After the experiment, the participants needed to fill out the S-AI.

### Results and discussion

As in experiment 1, the anxiety score of the second experiment was also the sum of the standard score for those reverse scoring statements.

**Fig. 5:**
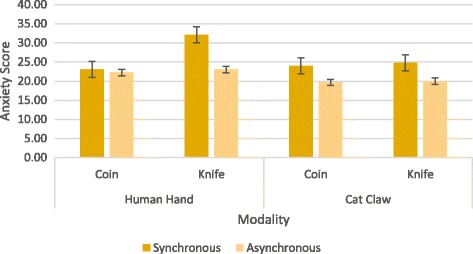
The results of experiment 2

The mean ratings for anxiety were submitted to a 2 × 2 × 2 ANOVA with the three factors which are synchronicity (synchronous vs. asynchronous), modality (human hand vs. cat claw), and event (catching coins vs. avoiding knives) (see Fig. 5). There were significant main effects of synchronicity, modality, and event (*F* (1, 95) = 32.62, *p* < 0.001, *η* ^2^_*p*_ = 0.41, *F* (1, 95) = 13.01, *p* = 0.001, *η* ^2^_*p*_ = 0.28, *F* (1, 95) = 10.60, *p* = 0.002, *η* ^2^_*p*_ = 0.25, respectively). The participants showed a stronger sense of anxiety for synchronous (*M* = 25.98, SD = 4.96) than asynchronous views (*M* = 21.23, SD = 4.65), a stronger sense of anxiety for the human hand (*M* = 25.10, SD = 5.65) than the cat claw (*M* = 22.10, SD = 4.61), and also a stronger sense of anxiety for avoiding knives (*M* = 24.96, SD = 6.62) than catching coins (*M* = 22.25, SD = 3.18). The results of main effects suggested the sense of controlling one’s own movement, the sense of ownership over the moving body part, and a threaten event can put more stress on an individual. The participants who needed to avoid knives in the synchronous human hand condition reported the highest anxiety through the questionnaire.

Besides, the interaction among synchronicity, event and modality was also significant (*F* (1, 95) = 5.54, *p* = 0.021, *η* ^2^_*p*_ = 0.57). Avoiding knives in synchronous human hand condition led the participants to gain the highest anxiety score (*M* = 32.08, SD = 3.655) while catching coins in asynchronous cat claw made the lowest anxiety score (M = 19.67, SD = 2.060).

The second experiment provided evidence for the interaction among sense of agency, sense of ownership, and emotional events. Overall, avoiding knives in synchronous human hand condition produced the highest anxiety score while catching coins in asynchronous cat claw had the lowest anxiety score. As in experiment 1, synchronicity and modality significantly affected anxiety, and there is no relevance to the type of the task. Besides that, the results also revealed that different emotional events influenced stress feelings differently. It was easier to arouse higher anxious feelings in the participants when they were assigned with the task of avoiding knives than when catching coins. That was due to the affective effects of emotional events on stress feelings. The ability of detecting, identifying, and avoiding threats are more important for our survival than gaining awards (Boyer & Bergstrom, 2011; Nairne & Pandeirada, 2016). Moreover, we also found both synchronicity and modality had more influences on punishing than on awarding, which suggested when the condition was taken as not so relevant to themselves, the corresponding anxiety level would substantially descent for threatening than awarding. There was also an interaction among sense of agency, sense of ownership, and emotional events, which fits with the claim that self-perception will be processed hierarchically with multimodal areas processing the confluence of “self” information from different sensory systems and explaining away the surprising incoming sensory information from unimodal areas (Apps & Tsakiris, 2014).

## Results and discussion

As two major components of minimal self, sense of agency and sense of ownership have attracted more and more study interests. These basic aspects of an individual’s self-perception of the body are critically important for self-consciousness, subjective embodiment, and self-other discrimination. The traditional paradigm of rubber hand illusion, because of its ability to create a sense of ownership over external effector that does not belong to the participant’s own body, has been a popular research method of the problem “self.” By adopting moving factor into traditional rubber hand illusion, moving rubber hand illusion (Kalckert & Ehrsson, 2012) or virtual hand illusion (Tsakiris, Prabhu, & Haggard, 2006) makes it possible to study both sense of agency and sense of ownership at the very same time. Previous studies focused more on the differences of sense of agency and sense of ownership or on which component is more fundamental; few researches paid attention to how these two basic experiences could affect individual’s emotional states. It is possible that they may even affect how we perceive the outside world or how we feel about or react to different emotional events, which contribute to series of physiological changes in the brain (Guterstam, Abdulkarim, &Ehrsson, 2015; Christensen, Yoshie, Di, & Haggard, 2016).

In the present study, we investigated how disentangled sense of agency and sense of ownership can interact with different emotional events on stress feelings. Taken together, our findings suggest four conclusions.

First, people experience more anxiety over virtual effector that looks more similar to their own hand. As we can tell from the results of the pilot study, the lack of similarity does not prevent people from experiencing agency, but it does lead to reduced anxiety even in synchronous conditions. This suggests our anxious feelings at least partially depend on our pre-existing internal representation of one’s own body shape. Such result is consistent with the claim that the experience of body ownership may represent a critical component of self-specificity as evidenced by the different ways in which multisensory integration in interaction with internal models of the body can actually manipulate important aspects of the self (Tskiris et al., 2010).

Second, people experience more anxiety over virtual effector that they perceive as if they can control. We systematically found increased anxiety scores in conditions with synchronous relationships between virtual images and real hands. As these conditions also increase the perception of sense of ownership, it makes sense to assume that bottom-up information (provided through synchrony) and top-down information (provided by modality) are integrated into a coherent percept (Zhang & Hommel, 2016). The sense of agency and sense of ownership as two major components of minimal self existing in the process of self-other recognition affect the higher level emotional experience via bodily self-perception (Zhang & Li, 2016).

Third, pronounced, systematic effects on anxiety are restricted to threat conditions. As compared to avoiding knives, catching coins produced rather low anxiety levels overall. This result fits with the framework effect and supports the theory that thinking about function is an important component of the survival processing effect proposed by evolutionary psychologists (Bell, Röer, & Buchner, 2015).

Fourth, modality affects women more than men while synchronicity affects men more than women. There is evidence to suggest that attributional style (AS) (it refers to the style an individual uses to explain previous positive and negative life events) and sense of agency/ownership are related. Baumeister and Brewer (2012) demonstrated a positive correlation between internal Locus of Control (attribution of the cause of life events to the self) and sense of agency. Men were more likely to believe they can control their action (“internals”) than women. Men respond most favorably to synchronicity with either the human hand or cat claw, and this favorable response is mediated by self-referencing. In contrast, women feel more sensitive about their own bodily modality which was reflected in the outside world (“externals”) either with synchronicity or not and only happen in the response to the human hand.

## Conclusions

The dissociation of sense of agency and sense of ownership is for sure, but how these two senses interact with each other is not clear enough. Data from behavioral experiments support the additive model, which indicates that sense of agency seems to promote the integration of the sense of ownership, pure sense of ownership is fragmentary, and sense of agency can integrate different body parts into a continuum and form a unified body awareness (Tsakiris, Prabhu, & Haggard, 2006; Synofzik et al, 2008; Stein, 1964). However, brain imaging experiments are more supportive of the independent model, according to which sense of agency and sense of ownership are essentially different experiences, triggered by different inputs, and using different brain networks, there is no direct overlap (Tsakiris, Longo, & Haggard, 2010). From our point of view, such inconsistency may lie in the experimental design. Since we usually experience sense of agency with sense of ownership, the role of these two types of experiences may not be separated in normal behavioral experiments. Through our research, we can find that when sense of agency and sense of ownership were properly separated, the results obtained are consistent with the hypothesis proposed by the independent model. However, our findings do not support claims that sense of agency and sense of ownership have no interactions. The interaction of modality and synchronicity had a significant impact on sense of ownership rather than sense of agency, indicating sense of agency seems to increase sense of ownership but not vice versa.

Besides the relationship between sense of agency and sense of ownership, our study can also provide inspiration for minimal self and narrative self. Some philosophers suggested important distinctions between minimal self, a self devoid of temporal, and narrative self, which involves personal identity and continuity across time (Gallagher, 2000a, b). As we explained above, our hypothesis is mainly based on minimal self which can be further divided into two components, sense of agency and sense of ownership. However, this research can also help us to understand the relationship between minimal self and narrative self. According to the results of the first experiment, both higher sense of agency and sense of ownership produced higher anxiety scores, which indicates that current experience or perception of our body will influence higher level cognition of emotion. Further, from the second experiment, we can see such effect only exists in the task of avoiding knives, which suggests cognition, bodily sense of self, and emotional experience are at least based on partly overlapping information. Taken together, these two experiments can be taken to indicate such a possibility that emotion is probably the key component for binding minimal self and narrative self (Medford, 2012). In fact, this idea can be tracked back to William James who proposed a similar standpoint in *The Principle of Psychology*. According to James, all types of self experience are accompanied by corresponding emotional representations, which are the fundamental elements of the self (James, 1890). Evidence from depersonalization disorder can be seen as one kind of support. However, we still need a large amount of systematic research to reveal the underling mechanism of minimal self and narrative self.

We only observed systematic effects on anxiety are restricted to plausible threats. Given that collecting coins is likely to induce some affect and arousal, it is possible that SCR measures would have been more sensitive to pick up affective processes in the coin task. Further studies would be better to adopt both questionnaire and objective measurements.
